# Detection delayed cerebral ischemia by qeeg in patients with acute SAH

**DOI:** 10.1186/2197-425X-3-S1-A780

**Published:** 2015-10-01

**Authors:** K Lapteva, O Sazonova, V Podlepich, E Troshina, I Savin, A Goryachev, E Sokolova, A Tyurina, V Vasil'chenko

**Affiliations:** Burdenko Neurosurgical Institute of Moscow, Moscow, Russian Federation

## Introduction

SAH- is one of the most common pathology in neurointensive care. There is a complication of SAH- cerebral vasospasm (CV), which contributes significant mortality and morbidity. CV is believed to be the origin of Delayed Cerebral Ischemia (DCI). There are different methods to diagnose DCI and CV: neurological exam, MRI, CT, TCD and angiography, but they can't detect ischemia on early stages of development. EEG is a noninvasive method, detects ischemia on early stages of its development, before clinical signs.

## Objectives

The aim of our study was to work out the most predictive qEEG indexes of DCI.

## Methods

We performed prospective pilot study from 9.2014 till 4.2015 year. 8 pts. in acute stage of SAH were included (15 recordings of EEG in total). The method of the study- qEEG. We performed EEG, TCD, CT and neurological exam the same day. The pts. with DCI were determined by CT. The duration of EEG recording was 60 minutes. We used standard software to get the values of indexes of qEEG: alpha/delta ratio-ADR (the power of rhythm in alpha range/the power of rhythm in delta range), alpha/theta ratio- ATR, alpha/beta ratio- ABR, theta/delta ratio-TDR. We collected Hunt-Hess scale grade at admission, data about the ischemia signs on CT, blood flow velocity (BFV) by TCD, the level of conscience on the moment of EEG recording.

## Results

We revealed significant difference in ADR, TDR in the groups of pts. with ischemia and without ischemia (ADR-(0,1(0,09;0,15)- no isch. and 0,04(0,03; 0,05)- isch., TDR -(0,32(0,31;0,38)- no isch. and 0,07(0,06; 0,15)- isch.), p < 0,05. We revealed significant difference in ADR, TDR between two groups of pts. with different level of consciousness: (ADR- 0,04(0,02;0,05)- stupor/sopor and 0,11(0,09;0,44)- consciousness; TDR- (0,06(0,05;0,15)- stupor/sopor and 0,35(0,28;0,45)- consciousness), p < 0,05. We revealed significant difference in ADR, TDR (p < 0,05) between groups of pts. with different severity by H&H scale (ADR -(0,04(0,02;0,05)- 3-4 H&H scale and 0,11(0,07; 0,14)- 1-2 H&H scale; TDR -(0,06 (0,05;0,15)- 3-4 H&H scale and 0,32(0,28;0,38)- 1-2 H&H scale). We revealed no significant difference in indexes of qEEG between groups of pts. with different BFV (< 120 sm/sec; 121-150 sm/sec; > 151 sm/sec).

## Conclusions

For detection DCI not only ADR can be used, but also TDR. We suggest, that DCI can lead to diffuse encephalopathy, that affect the level of consciousness, that is why we revealed significant difference in ADR, TDR between groups of pts. with different level of consciousness. The H&H scale is based on the idea that the level of consciousness reflect the level of ischemia. It's well known that TCD doesn't always detect CV, furthermore, CV doesn't always lead to DCI. That is why we didn't reveal significant difference in qEEG between groups of pts. with different BFV. The limitation of our study is small sample group of patients.

